# Definition of a tool to assess shared decision‐making (SDM) on women with breast cancer: A value‐based approach

**DOI:** 10.1002/hsr2.817

**Published:** 2022-09-13

**Authors:** Carmen Angioletti, Egidio de Mattia, Luca M. Carloni, Alisha Morsella, Alessandra Fabi, Armando Orlandi, Giampaolo Tortora, Antonio G. de Belvis

**Affiliations:** ^1^ Critical Pathways and Outcomes Evaluation Unit Fondazione Policlinico Universitario Agostino Gemelli IRCCS Roma Lazio Italy; ^2^ Scuola Superiore Sant'Anna Istituto di Management Pisa Toscana Italy; ^3^ Università di Pisa Lungarno Antonio Pacinotti Pisa Toscana Italy; ^4^ Università Cattolica del Sacro Cuore‐Campus di Roma Roma Lazio Italy; ^5^ Breast Precision Medicine Unit Fondazione Policlinico Universitario Agostino Gemelli IRCCS Roma Lazio Italy; ^6^ Comprehensive Cancer Center, UOC di Oncologia Medica Fondazione Policlinico Universitario Agostino Gemelli IRCCS Roma Lazio Italy

**Keywords:** breast cancer, patient‐reported outcomes measures, shared decision making, value‐based healthcare

## Abstract

**Background and Aims:**

In oncology, there is increasing talk of personalized treatment and shared decision‐making (SDM), especially when multiple treatment options are available with different outcomes depending on patient preference. The present study aimed to define the set of main dimensions and relative tools to assess the Value brought to patients from a Breast Cancer's Clinical pathway structured according to a dynamic SDM framework.

**Methods:**

Starting from our previous systematic review of the literature, a deep search of the main evidence‐based and already validated questionnaires was carried out. In the second phase, to corroborate this grid, a Delphi survey was conducted to assess each questionnaire identified for each dimension, against the following seven value‐based criteria: Clinical Benefit, Safety, Care Team Well Being, Patient Reported Outcomes Measures, Green Oncology, Impact on Health Budget, and Genomic Profile.

**Results:**

The resulting 7‐dimension questionnaire is composed of 72 questions. Of these, some quantitatively and objectively assess the evolution of the patient's disease state, whereas others aim to ask patients about their active involvement in decisions affecting them and to investigate whether they were free to explore their preferences. Furthermore, to frame the analyzed phenomenon at the right time, for each questionnaire section, the specific, evidence‐based timing of administration is indicated.

**Conclusion:**

The resulting questionnaire is validated in its entirety and it is composed of a set of questions and relative time point for data collections to assess the Value brought to patients undertaking a Breast Cancer's Clinical pathway, structured according to a dynamic SDM framework. It constitutes a quantitative instrument to integrate patient centeredness with a personalized perspective in the care management of women with breast cancer.

## 1 INTRODUCTION

Breast cancer is the second leading cause of female deaths due to cancer worldwide.^1^ In particular, at national level, it represents the most common type of cancer for women (30%), followed by colorectal cancer (11.2%), lung cancer (7.3%), thyroid cancer (5.4%), and endometrial cancer (4.6%).^2^ The incidence of breast cancer in Italy appears to slightly increase (+0.3% per year) on an annual base, while mortality continues to decline significantly (−0.8% per year). The country's 5‐year survival rate of women with breast cancer is 87%.^3^

Given the improvement in survival rates for breast cancer patients, attention to quality‐of‐life issues is becoming increasingly relevant. To deliver more consistent, safe, high quality, and evidence‐based care for people, breast cancer care should be organized according to its appropriate clinical pathway.

Clinical pathways can be defined as clinical governance tools capable of optimizing the spatial and temporal sequence of structured multidisciplinary care plans used by health services to detail essential steps in the care of patients with a specific clinical problem, with the goal of linking evidence to practice and optimizing clinical outcomes, while maximizing clinical efficiency^4^, ^5^, ^6^ For oncological care, this includes recognizing the first signals of cancer, conducting symptom‐based investigations, and going through the various diagnostic processes leading to diagnosis, treatment (surgery, radiation, or chemotherapy), as well as the posttreatment‐care programs.^7^

New organizational paradigm should be read in relation to the conceptual change that is contradicting modern healthcare organizations: from a vertical (specialty centered) organization to a horizontal (patient‐centered) process‐managed organization, for which increasingly innovative biomedical therapies are being discovered.^7^

To ensure patients are at the core of their own care process, beginning to measure, analyze, and improve outcomes during the delivery of care is a critical step. Therefore, the quality monitoring and improvement system, made up of key performance indicators, must be consolidated and ambitious, as it is generally the primary means of verification and measurement that ensures continuous quality of care improvement.^8^

However, due to the information asymmetry that characterizes the doctor–patient relationship, patient centeredness, patient empowerment, and a better relationship between the patient (and his family members) and the doctor can play a fundamental role in exploring the patient's preferences and values, monitoring his degree of satisfaction and helping him to make the right choices.

According to the definition coined by the Institute of Medicine, patient‐centered care means “Providing care that is respectful of and responsive to the preferences, needs, and values of individual patients, ensuring that patients' values guide all clinical decisions.” Indeed, the context of cancer treatment is particularly challenging for patients and their families, because multiple effective therapies are interconnected and there is a complex interplay between their benefits and risks.^9^, ^10^

This leads patients to participate in their health choices, taking an active role in expressing their concerns about data sharing and access to personalized treatments.^11^

The primary aims of shared decision‐making (SDM) in this context are realized when patients are fully informed of treatment choices in terms of risks and benefits, and when patient values and preferences are included into treatment decisions.^9^

Thus, one can clearly discern^10^ how participation in SDM is considered a keystone in the achievement of sustainable high‐quality cancer care, especially when several treatment options with similar overall potential may yield very different results depending on patient preferences.^12^ SDM has been defined as “an approach where clinicians and patients share the best available evidence when faced with the task of making decisions and where patients are supported to consider options, to achieve informed preferences.”^13^ Such activity aims at SDM is an approach where clinicians and patients make decisions together using the best available evidence, respecting patient autonomy and promoting patient engagement.^14^ Therefore, creating a tool to provide such information to physicians and patients should be a priority.

**Figure 1:**
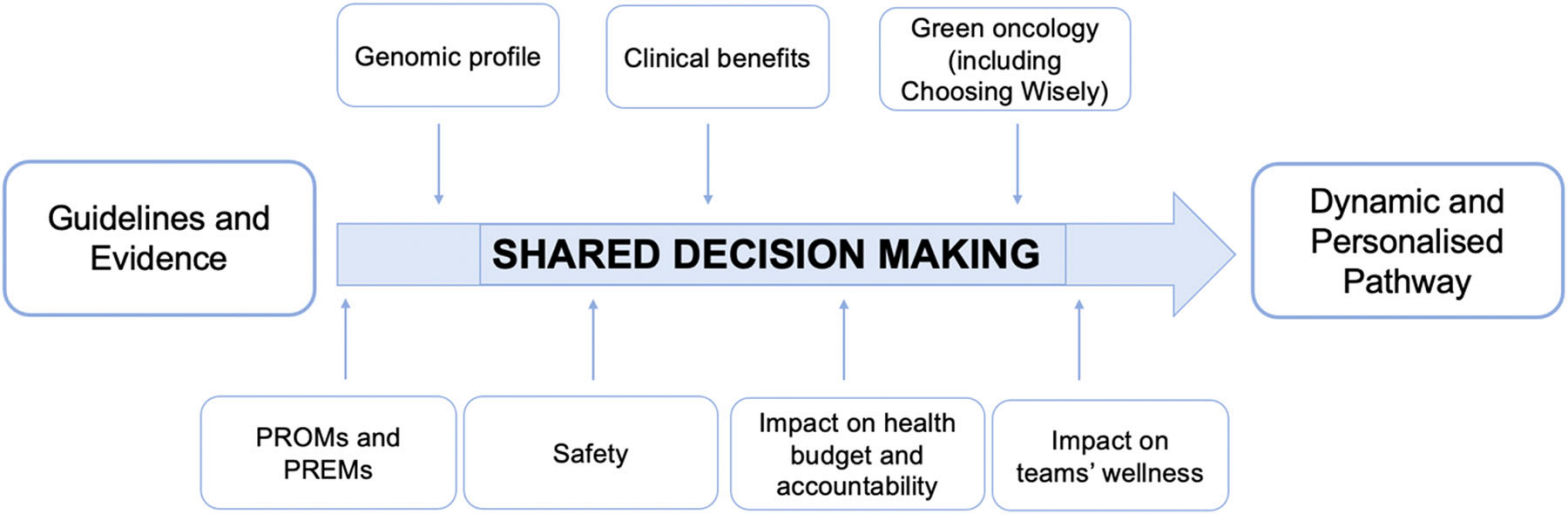
Shared Decision‐Making framework^18^

Thus, as a development of the existing value‐based assessment approaches,^15^, ^16^, ^17^ this study aims at applying the results of a previous systematic review from our research team (Figure 1),^18^ so as to provide a tool to assess value on women undertaking a breast cancer care pathway through an SDM perspective.

## 2 METHODS

To assemble the appropriate set of questions, the authors adopted a methodology divided into two phases.

First of all, building on the findings of a previous systematic review,^18^ an extensive search of the main evidence‐based and already validated questionnaires in the literature was carried out.

The main databases (PubMed, Scopus, and Web of Science) and official websites of institutions and organizations with specific expertise in this field (Associazione Italiana di Oncologia Medica [AIOM], Collegio Italiano dei Primari Oncologi Medici Ospedalieri [CIPOMO], European Organization for the Research and Treatmentof Cancer [EORTC], International Consortium for Health Outcomes Measurement [ICHOM], and Istat) were consulted.

Subsequently, each questionnaire was analyzed to extract the items relevant to assessing the value brought to the patient by the BC clinical pathway. Precisely, both the contextual questions and the questions specific to the SDM were made explicit for each questionnaire. In addition, gaps in which the literature search produced scarce or irrelevant results were filled by consulting national and international guidelines.

The identification of the questionnaires was followed by the identification of the determined time points for data collection (identified in relation to the different steps of the clinical path specific to the patient with malignant breast cancer). Where no scientific evidence was found in the literature, timings were submitted to Delphi surveys^19^ (the second phase of our methodology).

In the second phase, to corroborate the result obtained and to make sure that the questionnaire indeed constitutes a recommendable tool, a two‐round Delphi survey was carried out.

Experts among healthcare professionals (two breast surgeon, two breast medical oncologists, one case manager, two nurses, one geneticist, one palliative therapist, and one postgraduate training doctor in public health), academic experts (one manager and three economists), and “expert patient” were invited to assess each questionnaire identified for each dimension against the following four criteria:

- General relevance

- Support from scientific evidence
- Measurability
- Actionability.

The team of experts was invited to complete the Delphi survey by email, through a Google Modules questionnaire. In particular, a cover letter explained the purpose, relevance, and usefulness of this survey. The answers were collected immediately and anonymously.

This methodology replicated one already applied by the team to another clinical setting.^20^

For Delphi's first round, experts were asked to express their degree of agreement on a Likert scale from 1 to 3 (with 1 corresponding to the lowest—“Not relevant” and 3 to the highest—“Relevant”), with the set of the statements formulated for each question, with regard to the four criteria described above.

The first round of consultation started on June 22, 2021, and ended on June 30, 2021.

The following levels of agreement were considered:

- *“*Strong agreement”: “Overall” score of the item is ≥2.5 out of 3.0.
- “Agreement for exclusion”: “Overall” score for each item is <2.0 out of 3.0.

In the presence of a “strong agreement for inclusion,” the question was included in the second round of the survey. Items falling in the category “agreement for exclusion” were eliminated.

Delphi's second round was structured as the first one. For the final list of questions, the following levels of agreement were established:

- *“*Strong agreement for inclusion in the final list”: “Overall” score ≥2.5 out of 3.0.
- “Agreement of exclusion from final list”: mean of “Overall” score for each item <2.0 out of 3.0.

The second round of consultation started on July 1, 2021, and ended on July 7, 2021.

## 3 RESULTS

### 3.1 Literature search results

In the first phase, an extensive literature review was conducted to analyze in detail each of the items depicted in the figure (Figure 1). Based on the review's results, only questionnaires designed to be administered to patients and with proven effectiveness in the relationship between data collection and improvement in the quality of care perceived by the patient were considered.

Below are the main sources identified:

1) EORTC QLQ‐C30^21^ Systematic Survey of Patient Experience and Outcomes in the Tuscan Health Care System^22^ and AIOM Guidelines PCA Lazio 2021 for Clinical Benefit;
2) Decisional Conflit Scale,^23^ FLOW‐CHART, Guidelines and iSHAREpatient^24^ or Safety;
3) CollaboRATE^25^ for Patient Reported Experience Measures (PREMs) and Care Team Well Being;
4) BREAST‐Q^26^ and EORTC QLQ‐BR23^27^ for Patient Reported Outcome Measures (PROMs);
5) Guidelines for Green Oncology;
6) Istat Multiscopo or modified Istat Multiscopo for Impact of Health Budget;
7) Flow‐Chart for Genomic Profile.

### 3.2 Delphi results

#### 3.2.1 First round of consultation

Nine (60%) out of the 15 experts recruited responded to the first round.

A summary table, representative of the mean of all the Excel files received, is shown below. The analytical results are reported below by question and by evaluation criterion (Tables 1, 2, 3, 4, 5, 6, 7, 8).

**Table 1:** Delphi first and second round—Introduction

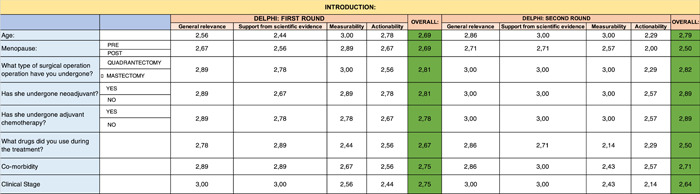

**Table 2:** Delphi first and second rounds—Clinical Benefit section

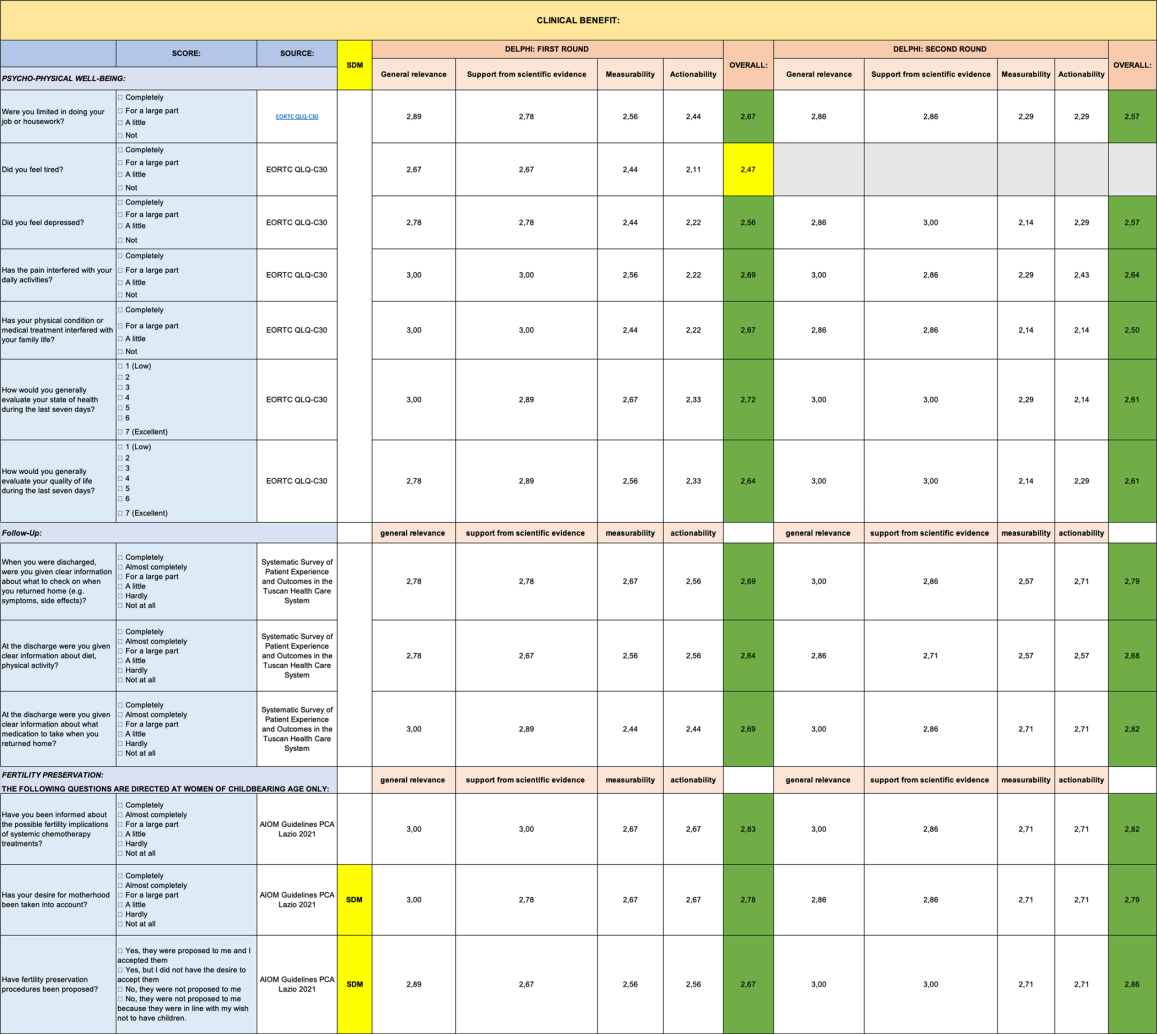

**Table 3:** Delphi first and second rounds—Safety section

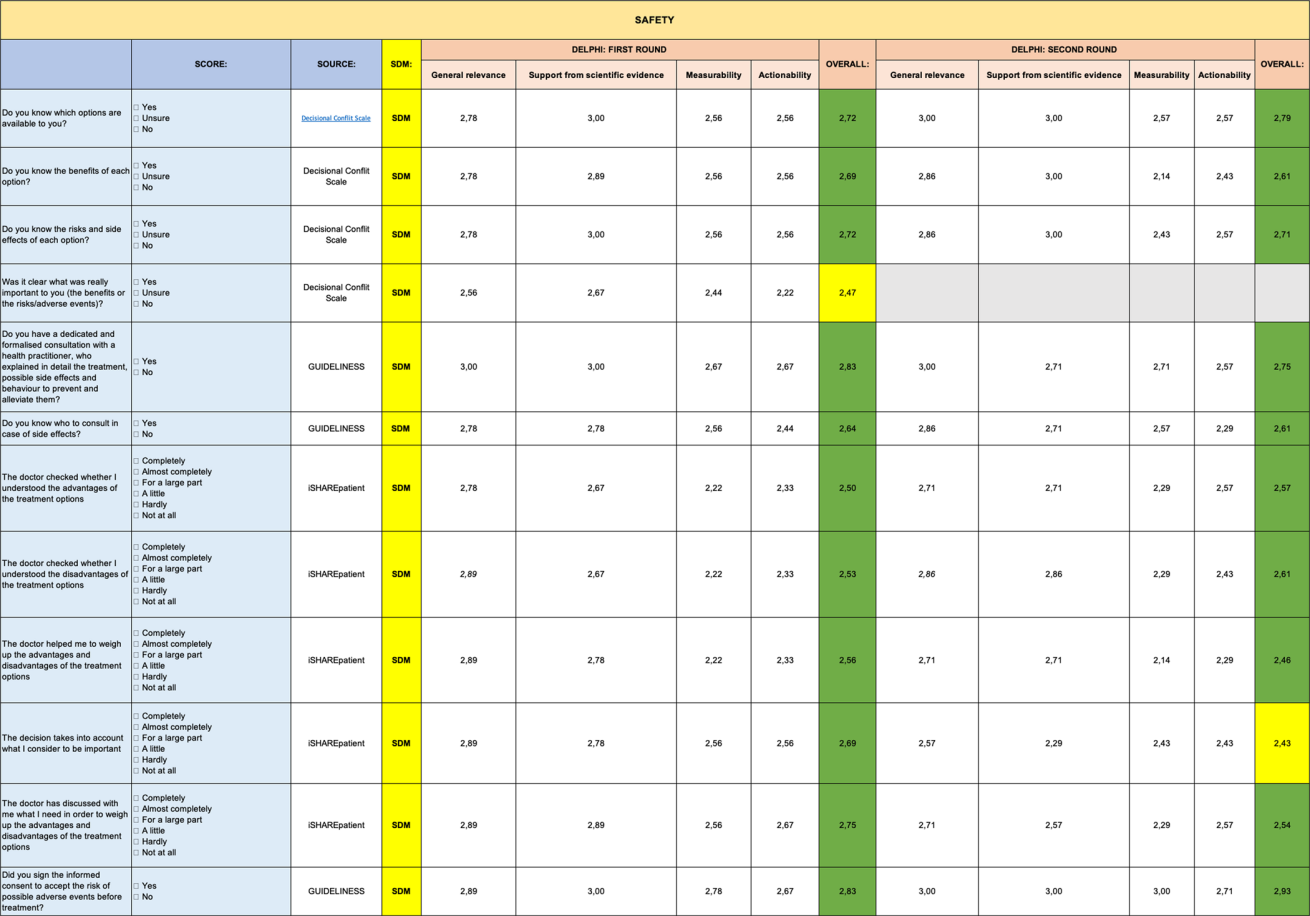

**Table 4:** Delphi first and second rounds—Care Team Well Being section

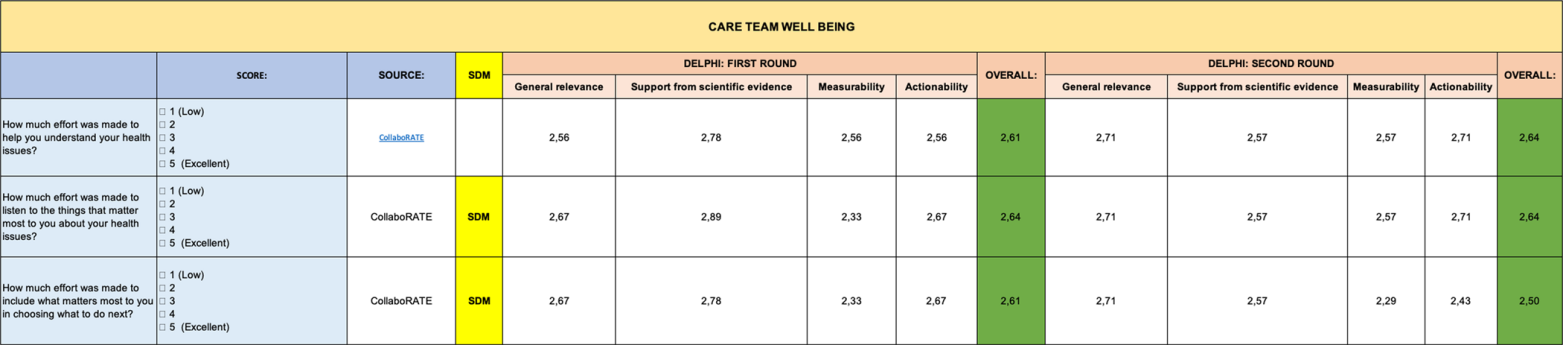

**Table 5:** Delphi first and second rounds—PROMs section

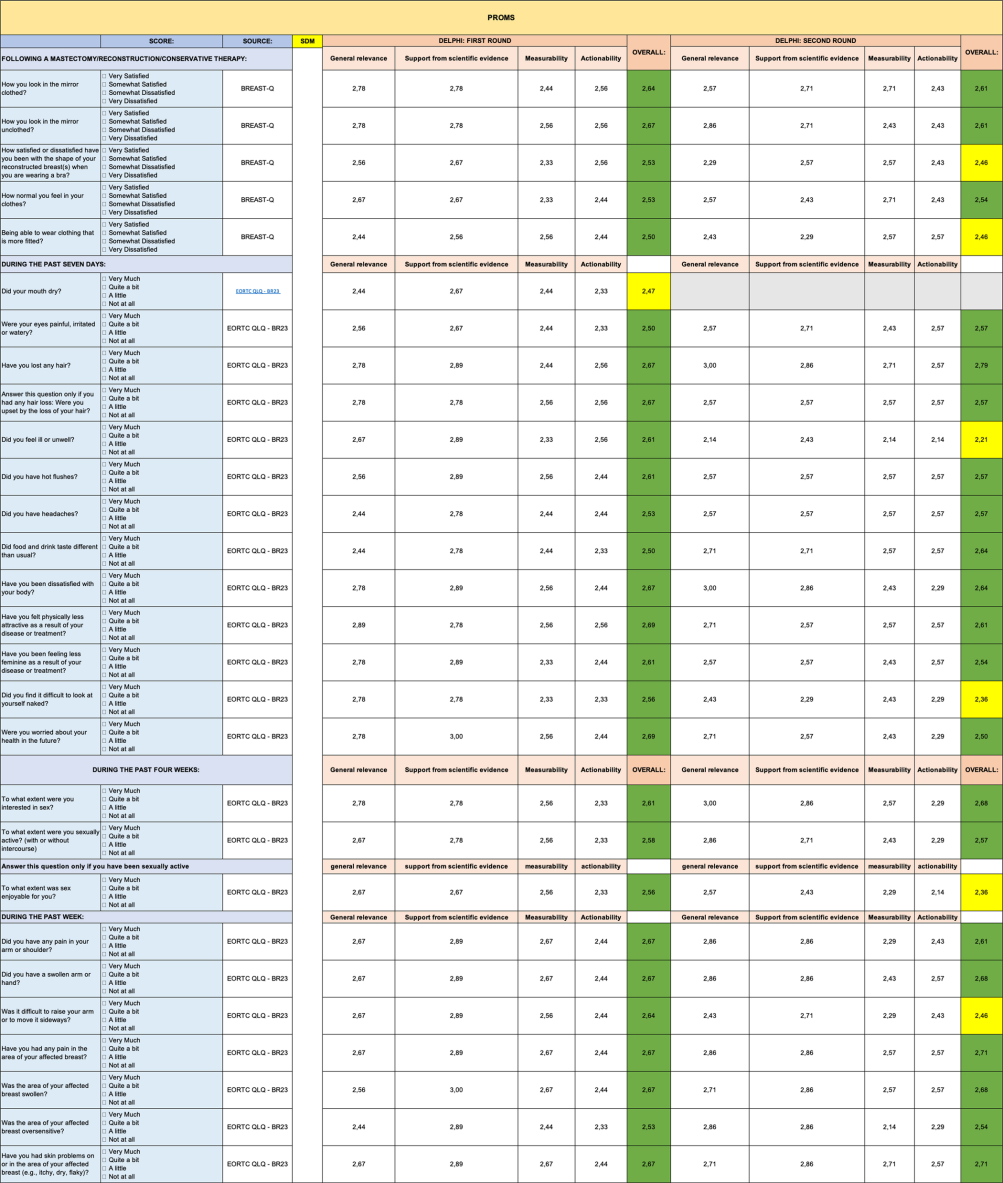

Abbreviation: PROMs, Patient Reported Outcome Measures.

**Table 6:** Delphi first and second rounds—Green Oncology section

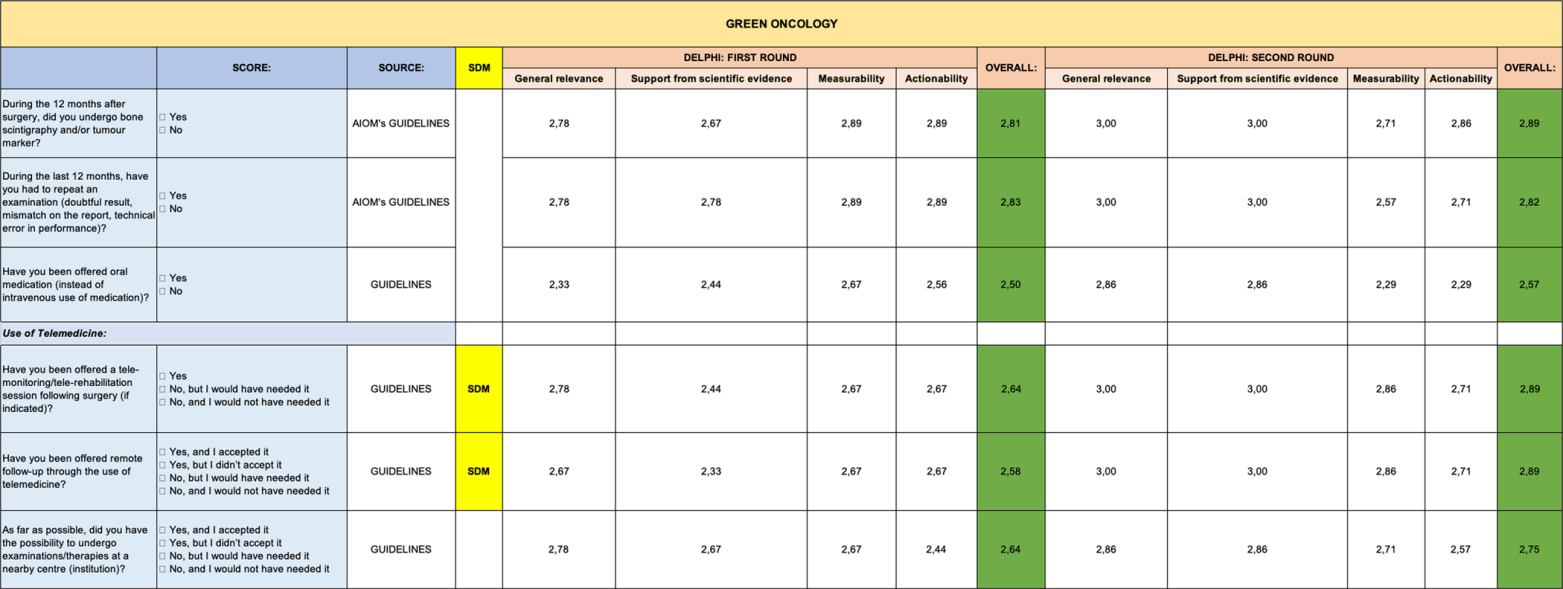

**Table 7:** Delphi first and second rounds—Impact of Health Budget section

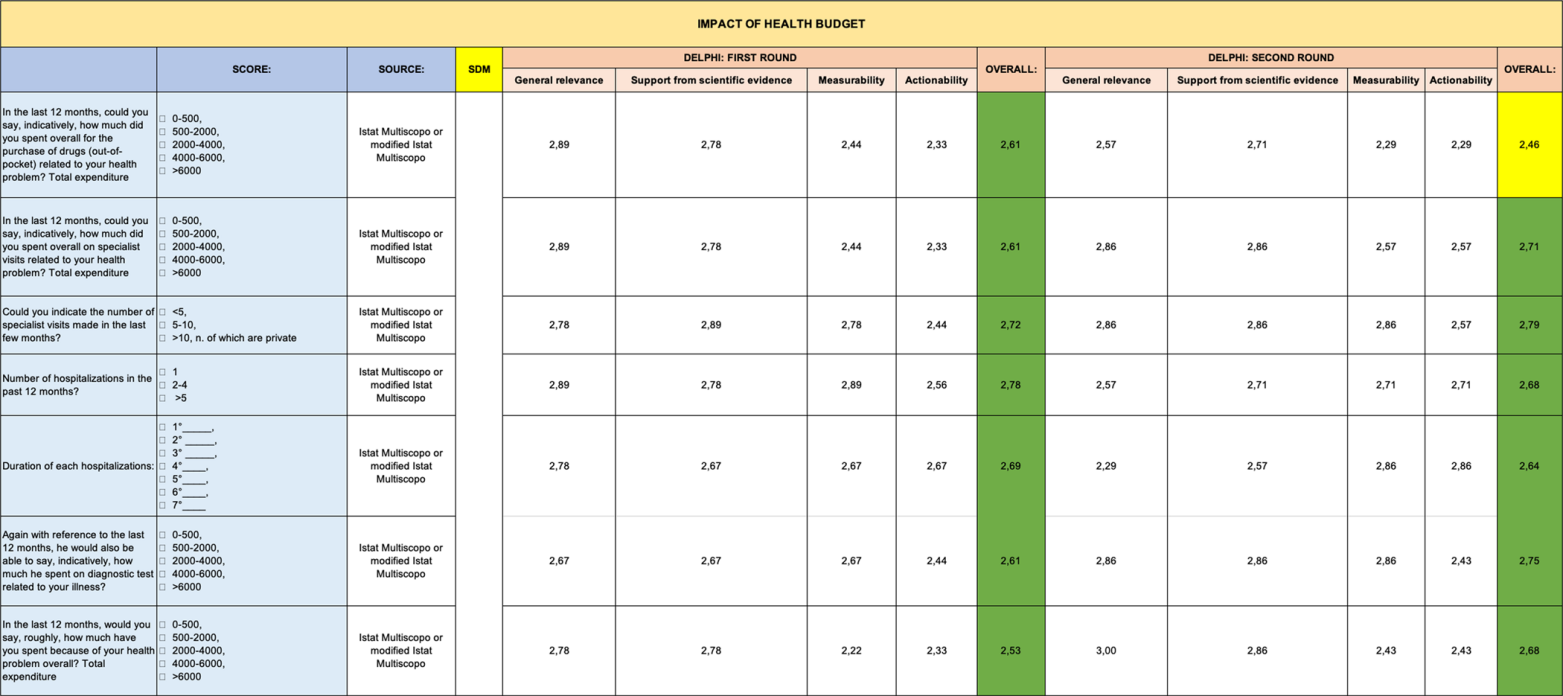

**Table 8:** Delphi first and second rounds—Genomic Profile section

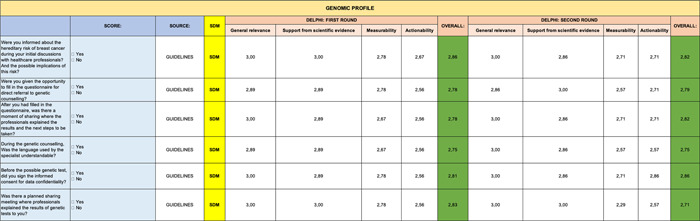

Only 3 questions out of 84 (0.04%) received an average rating of less than 2.5 per evaluation criterion and were therefore not considered for second round of consultation.

Specifically, these three entries came from the following sections: Clinical Benefit (Table 2), Safety (Table 3), and PROMs (Table 5).

In contrast, the remaining sections (Genomic Profile [Table 8], Impact of Health Budget [Table 7], Green Oncology [Table 6], and Care Team Well Being [Table 4]) were validated entirely in first round. However, the PROMs section (Table 5) received the lowest scores on some items.

On the other hand, with regard to administration times that had not been previously validated in the literature, the proposed times were all accepted by the experts. Specifically, though, experts proposed other solutions when it came to the Impact of Health Budget section and they were included for validation in the second consultation round.

#### 3.2.2 Second round of consultations

Seven (77.8%) out of the nine experts recruited responded to the second round.

The resulting 7‐dimension questionnaire is composed of 72 questions (8 of the latter belong to “Introduction” section) (Figure 1).

The PROMs section (Table 5) shows the greatest reduction in items during second round. Six out of eight items eliminated during the second round of the Delphi analysis came from the following section. The other two items eliminated came from the Safety (Table 3) and Impact of Health Budget (Table 7) sections.

The experts considered all items of the Clinical Benefits section (Table 2) suitable, although the measurability score had the lowest score of the second round. On the other hand, we were able to maintain the validity of the Genomic Profile (Table 8) and Care Team Well Being (Table 4) sections in their entirety.

It is interesting to note how the items of the Green Oncology (Table 6) section report higher ratings in the second round than in the first one.

A summary table, representative of the mean of all the Excel files received, is shown below. Indicators represented in green are those which reached positive evaluations for inclusion with “strong agreement”; in yellow, instead, are those which reached a “partial agreement” (items with a score equal to or >2.0 out of 3.0). No indicator scored lower than 2.0, which would have implied exclusion from the final list.

**Figure 2:**
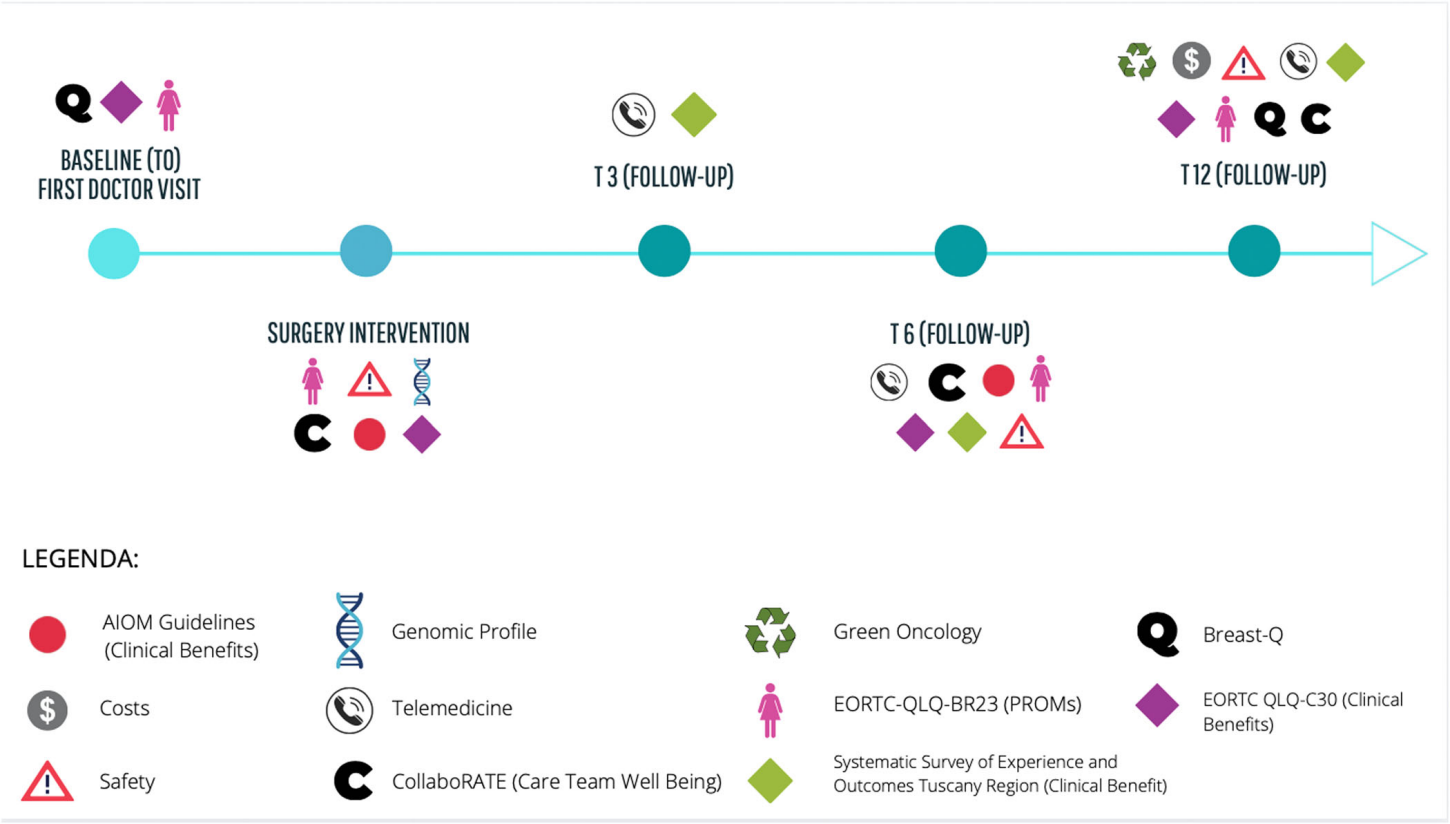
Time points for data collection

The survey then allowed us to identify the evidence‐based timing of administration related to each section of the questionnaire, to capture the phenomenon under analysis at the right time (Figure 2).

## 4 DISCUSSION

Our study aimed to define a tool consisting of a set of questions and relative timing to assess the value brought to patients undertaking a Breast Cancer's Clinical pathway, structured according to a dynamic SDM framework.

We applied the evidence from a previous systematic review of our research team that introduced a new framework to measure the level of SDM among women treated for breast cancer, including seven dimensions: Genomic Profile; PREMs and PROMs; Safety; Clinical Benefits; Green Oncology; Impact on the Health Budget and Accountability; Impact on Team Wellness, to measure the level of SDM among women treated for breast cancer (Figure 1).^18^

Such a quantitative assessment tool (Figure 2) was set up by considering which questions—for each of the value‐based dimensions—should be administered to the patient and their relative timing of administration in the different episodes of the care pathway.

The resulting 7‐dimension questionnaire is composed of 72 questions. Of these, some quantitatively and objectively assess the evolution of the patient's disease state (e.g., Have you had any pain in the area of your affected breast?), whereas others aim to ask patients about their active involvement in decisions affecting them and to investigate whether they were free to explore their preferences (e.g., The doctor helped me to weigh up the advantages and disadvantages of the treatment options).

Once administered, this dynamic tool, based on the patient's perspective, would generate “cumulative scores” and allow to evaluate:

1. The course of the patient's clinical condition and quality of life over time or within a CP;
2. Homogeneous groups of patients over time;
3. The performance of each professional;
4. The safety (compliance) of treatments;
5. The economic, social and environmental impact of the Care Pathway' from the patient's perspective.

This tool portrays a comprehensive landscape of the SDM implementation trend and, on the other hand, serves to verify if the results achieved at the level of each question comply with evidence‐based standards. Also, the establishment of “minimum thresholds” will allow clinicians to activate improvement interventions (audit) in case of overruns.

In recent years, many studies have focused on the concept of value in healthcare and the methodologies that organizations should/could use to assess it. However, to evaluate the theme of the patient's experience with health care, methodologies traditionally focus mainly on the analysis of PREMs and PROMs. As PROMs aims to monitor the impact of a given treatment, their joint combination focuses on surveying a more patient‐centered approach to care, by providing the patients' view along the whole pathway.

As for PROMs questionnaires, an increasing use in clinical practice has been registered over time. Thus, we adopted PROMS, an internationally recognized tool for assessing the quality of care during different care pathway's steps and benchmarking among providers, as worldwide recommended by the ICHOM.^28^, ^29^, ^30^, ^31^

Several systematic reviews have been conducted with the goal of identifying which major PROMs questionnaires have been developed specifically for patients with breast cancer facing different stages of disease and treatments.^32^, ^33^, ^34^ An interesting example is the work realized by van Egdom et al.^32^ This review investigated the implementation methods, impact facilitators, and barriers of PROM collection in breast cancer clinical practice. However, as reported previously, our paper is not a systematic review of existing PROMs questionnaires used in the Breast Cancer clinical practice. With our work, we aimed to take a step forward and to create a comprehensive tool capable of investigating the main dimensions that characterize SDM in the Breast Cancer clinical practice according to the patient's perspective. Consequently, the PROMs questionnaires represent only one of the seven dimensions we investigated.

CollaboRATE^35^ and SURE^36^ are two important contributions in this regard. CollaboRATE focuses on patients' perceptions of being informed and then involved in decision‐making, whereas SURE focuses on patients' perceptions of conflicts in the decision‐making process.^25^ Thus, our study is part of this trend and aims to take a step forward in the same direction.

The most important strength of the study is the definition of a validated and replicable tool. It consists of seven dimensions, structured according to a dynamic SDM framework, including PREMs and PROMs, which the latest publications in the scientific literature emphasize are key elements for improving the quality of breast cancer management. Each dimension is composed of a set of questions to assess the value brought to patients undertaking a Breast Cancer Clinical pathway. It constitutes a quantitative instrument to integrate patient‐centeredness with a personalized perspective in managing women with breast cancer care.

In addition, as it is essential to understand when the questions should be administered to investigate the phenomenon under analysis in the best possible way, another interesting study result concerns the questionnaire's administration time. Each question/section is characterized by its relative time point for data collection in the different episodes of the care pathway.

Within this context, although, it is important to consider our findings in light of three main limitations:

– The assessment questionnaire investigates the patient's perspective, which is a subjective perspective.
– The Delphi methodology is characterized by its own intrinsic limitations. The starting material provided and the questions may not be representative; the process tends to eliminate extreme positions and force a middle‐of‐the‐road consensus; the outcome obtained is highly sensitive to the characteristics of the project, such as expertise and composition of the panel, clarity of the questions, and is vulnerable to high dropout rates due to the long time commitment required, distractions between rounds, or disillusionment with the process.^8^, ^37^
– There is a need to weigh the items' questions.

## 5 CONCLUSIONS

The resulting SDM evaluation tool is validated in its entirety and can provide a complete overview of the Value created by the clinical pathway for women with breast cancer. It constitutes a quantitative instrument to integrate patient‐centeredness with a personalized perspective in the care management of women with breast cancer.

Further developments of this study stream will be conducted by a pilot study assessment on critical pathways dedicated to women with breast cancer in our Research Hospital.

Our tool also lends itself to further adaptations and is modifiable and applicable for analyzing other critical pathways. Further studies are needed to demonstrate whether SDM tools can improve adherence and deeper patient involvement at critical points in their care pathway.

## AUTHOR CONTRIBUTIONS

**Carmen Angioletti**: Conceptualization; Formal analysis; Methodology; Writing–original draft; Writing–review & editing. **Egidio de Mattia**: Conceptualization; Formal analysis; Methodology; Writing–original draft; Writing–review & editing. **Luca M. Carloni**: Data curation; Validation. **Alisha Morsella**: Writing–review & editing. **Alessandra Fabi**: Data curation; Supervision. **Armando Orlandi**: Data curation; Validation. **Giampaolo Tortora**: Data curation; Validation. **Antonio G. de Belvis**: Conceptualization; Formal analysis; Supervision; Validation; Writing–original draft.

## CONFLICT OF INTEREST

The authors declare no conflict of interest.

## ETHICS STATEMENT

All procedures performed in studies involving human participants were by the Institutional and National Research Committee's Ethical Standards and the 1964 Helsinki Declaration and its later amendments or comparable ethical standards.

## TRANSPARENCY STATEMENT

The lead author Egidio de Mattia affirms that this manuscript is an honest, accurate, and transparent account of the study being reported; that no important aspects of the study have been omitted; and that any discrepancies from the study as planned (and, if relevant, registered) have been explained.

## Data Availability

The authors confirm that the data supporting the findings of this study are available within the article and/or its supporting information.
